# Arginine Is a Critical Substrate for the Pathogenesis of *Pseudomonas aeruginosa* in Burn Wound Infections

**DOI:** 10.1128/mBio.02160-16

**Published:** 2017-03-14

**Authors:** Jake Everett, Keith Turner, Qiuxian Cai, Vernita Gordon, Marvin Whiteley, Kendra Rumbaugh

**Affiliations:** a Department of Surgery, Texas Tech University Health Sciences Center, Lubbock, Texas, USA; b Department of Molecular Biosciences, Institute of Cellular and Molecular Biology, John Ring LaMontagne Center for Infectious Disease, The University of Texas at Austin, Austin, Texas, USA; c Department of Physics, the University of Texas at Austin, Austin, Texas, USA; d Monsanto Company, Chesterfield, Missouri, USA; UT Southwestern Medical Center Dallas

## Abstract

Environmental conditions affect bacterial behavior and can greatly influence the course of an infection. However, the environmental cues that elicit bacterial responses in specific infection sites are relatively unknown. *Pseudomonas aeruginosa* is ubiquitous in nature and typically innocuous. However, it is also one of the most prevalent causes of fatal sepsis in burn wound patients. The aim of this study was to determine the impact of environmental factors, specifically the availability of arginine, on the pathogenesis of *P. aeruginosa* in burn wound infections. Comparison of burned versus noninjured tissue revealed that l-arginine (l-Arg) was significantly depleted in burn wounds as a consequence of elevated arginase produced by myeloid-derived suppressor cells. We also observed that l-Arg was a potent chemoattractant for *P. aeruginosa*, and while low concentrations of l-Arg increased *P. aeruginosa*’s swimming motility, high concentrations resulted in diminished swimming. Based on these observations, we tested whether the administration of exogenous l-Arg into the burn wound could attenuate the virulence of *P. aeruginosa* in thermally injured mice. Administration of l-Arg resulted in decreased *P. aeruginosa* spread and sepsis and increased animal survival. Taken together, these data demonstrate that the availability of environmental arginine greatly influences the virulence of *P. aeruginosa in vivo* and may represent a promising phenotype-modulating tool for future therapeutic avenues.

## IMPORTANCE

Despite our growing understanding of the pathophysiology of burn wounds and the evolution of techniques and practices to manage infections, sepsis remains a significant medical concern for burn patients. *P. aeruginosa* continues to be a leader among all causes of bacteremic infections due to its tendency to cause complications in immunocompromised patients and its ubiquitous presence in the hospital setting. With the unforgiving emergence of multidrug-resistant strains, it is critical that alternative strategies to control or prevent septic infections in burn patients be developed in parallel with novel antimicrobial agents. In this study, we observed that administration of l-Arg significantly reduced bacterial spread and sepsis in burned mice infected with *P. aeruginosa*. Given the safety of l-Arg in high doses and its potential wound-healing benefits, this conditionally essential amino acid may represent a useful tool to modulate bacterial behavior *in vivo* and prevent sepsis in burn patients.

## INTRODUCTION

Nosocomial burn wound infections are responsible for up to 75% of deaths in thermally injured patients, and sepsis, secondary to infection, is the primary predictor of mortality (1–3). Mortality rates due to Gram-negative sepsis are over 60%, and the opportunistic pathogen *Pseudomonas aeruginosa* has the highest mortality rate among all causes of bacteremia (4). *P. aeruginosa* is equipped with a battery of virulence factors that help facilitate colonization of the burn wound and translocation through the vasculature and into the bloodstream (5–7); however, it is still unclear what instigates this rapid transition into the bloodstream.

The unchecked propagation and spread of *P. aeruginosa* are largely the consequence of impaired immunity. The host immune response that follows severe thermal trauma is a dichotomy of pro- and anti-inflammatory phases that results in a general state of immune dysfunction in burn patients. The early postburn cytokine response is largely characterized by the production of proinflammatory cytokines interleukin-1 (IL-1), IL-6, tumor necrosis factor alpha (TNF-α), and prostaglandin E2 (PGE2), causing massive inflammation that can transition to severe inflammatory response syndrome, ultimately resulting in shock and end organ failure (7, 8). The late postburn phase is dominated by the production of the anti-inflammatory cytokines IL-10 and IL-4 to compensate for the initial proinflammatory response (7, 9–11). If the burn covers more than 30% total body surface area (TBSA), the anti-inflammatory response can be so dramatic that the patient becomes severely immunocompromised, stemming from the activation of suppressor cell phenotypes, including CD4^+^ CD25^+^ regulatory T cells and a subset of immunoregulatory cells termed myeloid-derived suppressor cells (MDSCs) (12–16).

MDSCs can suppress both adaptive and innate immune responses in part by sequestering critical substrates necessary for T cell activation and proliferation. For example, MDSCs can respond to high levels of inflammation by increasing production of arginase I (ArgI), which subsequently depletes the environment of available arginine, thus reducing protein synthesis (17). Limited availability of arginine results in T cells being arrested in the G_0_-G_1_ phase of the cell cycle, preventing effective activation and proliferation and crippling the immune system from an adaptive defense standpoint (18).

Furthermore, severe burn trauma causes devascularization of the wound environment, which prevents neutrophils and other innate effector cells from migrating into the wound and clearing the bacterial infection. The avascular state of the burn wound results in a decreased supply of oxygen and other essential nutrients to the damaged tissue, which can lead to ischemia (19). Despite these limitations, *P. aeruginosa* can flourish within the burn wound and aggressively disseminate through the surrounding tissue and into the bloodstream. Reports have indicated that the availability of key amino acids, especially arginine, can influence the lifestyle and activity of *P. aeruginosa* within the cystic fibrosis (CF) lung environment by acting as an environmental cue (20, 21). In this study, we investigated the impact of arginine availability in a thermally injured mouse model of infection to assess whether limited access to this substrate influences the behavior of *P. aeruginosa* pathogenesis in burn wound infections.

## RESULTS

### Depleted arginine levels are associated with concentrated MDSC recruitment at the burn wound.

**FIG 1:**
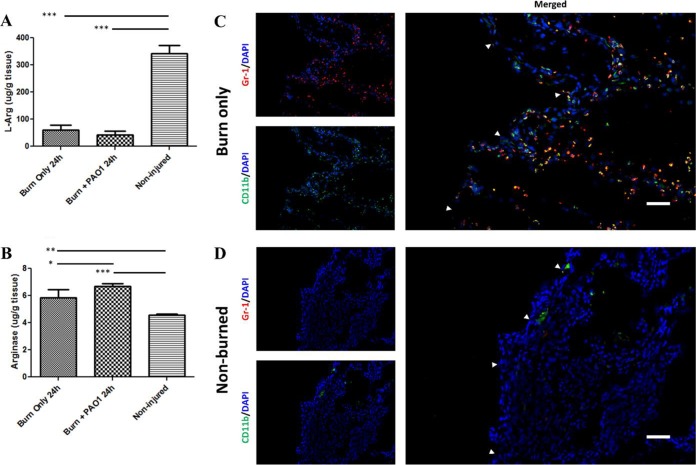
Depleted arginine levels are associated with MDSC recruitment. Tissue from mice that received thermal insult (with or without infection) was harvested 24 h post-burn injury. (A) l-Arg concentrations were significantly reduced in burned mice (with or without PAO1 infection) compared to the native concentrations of l-Arg in noninjured tissue by one-way analysis of variance (ANOVA) with Newman-Keuls multiple comparison posttest. ***, *P* < 0.001 (*n* = 5 mice/group). (B) Arginase concentrations were significantly elevated in tissue from mice that received a thermal insult or insult coupled with PAO1 infection compared to basal arginase levels in noninjured tissue by one-way ANOVA with Newman-Keuls multiple comparison posttest. ***, *P* < 0.001; **, *P* < 0.01; *, *P* < 0.05 (*n* = 5 mice/group). Tissue was harvested from (C) burn-only mice 24 h post-thermal insult or (D) nonburned mice. Tissue samples were prepared for direct immunofluorescence microscopy with FITC-labeled anti-CD11b and PE-labeled anti-Gr-1 antibodies. Host cell nuclei were counterstained with DAPI (blue). (C) A large number of MDSCs coexpressing both CD11b and Gr-1 are recruited to the burned tissue-intact tissue interface (white arrows). (D) No MDSCs were observed in the tissue from the dorsum of nonburned mice. Sections were visualized via a Nikon Plan Fluor 20×/0.75 objective. White size bars represent 50 µm.

Previous studies have demonstrated a role for arginine in modulating *P. aeruginosa* behavior, including aspects of motility, which can subsequently influence *P. aeruginosa* virulence (20). Therefore, we measured the concentration of l-Arg in uninfected wounds of thermally injured mice and compared it to the concentration of l-Arg in nonburned tissue. *P. aeruginosa* can be detected in the bloodstream as early as 24 h after burn injury and infection; therefore, any significant change in arginine levels that would incur an effect on *P. aeruginosa* pathogenesis would occur at or before this time point. To our surprise, we observed that thermal insult alone caused an ~80% decrease (*P* < 0.001) in l-Arg levels by 24 h postburn, compared to the basal levels of l-Arg in nonburned tissue (Fig. 1A). Levels of arginase, the host enzyme that converts arginine to ornithine and urea, within these uninfected wounds increased ~30% (*P* < 0.01) as a result of burn injury, which could in part explain the reduction in l-Arg in these tissues (Fig. 1B).

As *P. aeruginosa* is capable of metabolizing arginine to generate ATP under anaerobic conditions (22, 23), we next investigated how the presence of *P. aeruginosa* impacted the levels of arginine and arginase in thermally injured tissues. Our results revealed that infection of burn wounds with *P. aeruginosa* did not significantly alter the levels of arginine (Fig. 1A), although the levels of arginase were slightly elevated (Fig. 1B). Collectively, these data indicate that whether infected or uninfected, thermally injured tissue possesses significantly less arginine and significantly more arginase than uninjured tissue (Fig. 1).

As MDSCs are well known to produce arginase I in response to inflammation, we hypothesized that these cells are responsible for the high levels of arginase, and subsequently low levels of arginine, in thermally injured tissue (17, 18). While some MDSCs are known to be present in thermally injured tissue (16), it is unclear whether MDSCs are generally recruited to the inflamed tissue or if MDSCs exhibit a propensity to accumulate at the wound margin. To visualize the recruitment and distribution of MDSCs to uninfected burn wounds, we performed immunofluorescence microscopy on intact tissue immediately adjacent to the burn wound. Differentiation of MDSCs from other lymphocytes was accomplished by probing with two fluorophore-conjugated antibodies specific for the cell surface markers Gr-1 and CD11b, which are uniquely coexpressed on MDSCs. We observed a substantial number of Gr-1^+^ CD11b^+^ cells recruited to the tissue bordering the wound by 24 h post-thermal insult, and the distribution of MDSCs was heavily concentrated at the wound margin (Fig. 1C; see Fig. S1 in the supplemental material). No MDSCs were observed in tissue from nonburned mice (Fig. 1D; Fig. S1), reinforcing the tenet that these cells are only recruited to environments associated with high levels of proinflammatory cytokines. Taken together, these data indicate that MDSCs are recruited to thermally injured tissue and suggest that this is the likely mechanism of arginine depletion in burn wounds.

10.1128/mBio.02160-16.1 FIG S1  MDSC recruitment in thermally injured mice. Tissue was harvested from burn-only mice 24 h post-thermal insult or nonburned mice. Tissue samples were prepared for direct immunofluorescence microscopy with FITC-labeled anti-CD11b (green) and PE-labeled anti-Gr-1 antibodies (red). Host cell nuclei were counterstained with DAPI (blue). A large number of MDSCs coexpressing both CD11b and Gr-1 are recruited to the burned tissue-intact tissue interface. No MDSCs were observed in the tissue from the dorsum of nonburned mice. Sections were visualized via a Nikon Plan Fluor 20×/0.75, 40×/1.30 oil, and 100×/1.30 oil objectives. White size bars represent 50 µm. Red size bars represent 20 µm. Download FIG S1, PDF file, 0.3 MB. Copyright © 2017 Everett et al. 2017 Everett et al. This content is distributed under the terms of the Creative Commons Attribution 4.0 International license.

### *P. aeruginosa* swimming motility is reduced by l-Arg.

**FIG 2:**
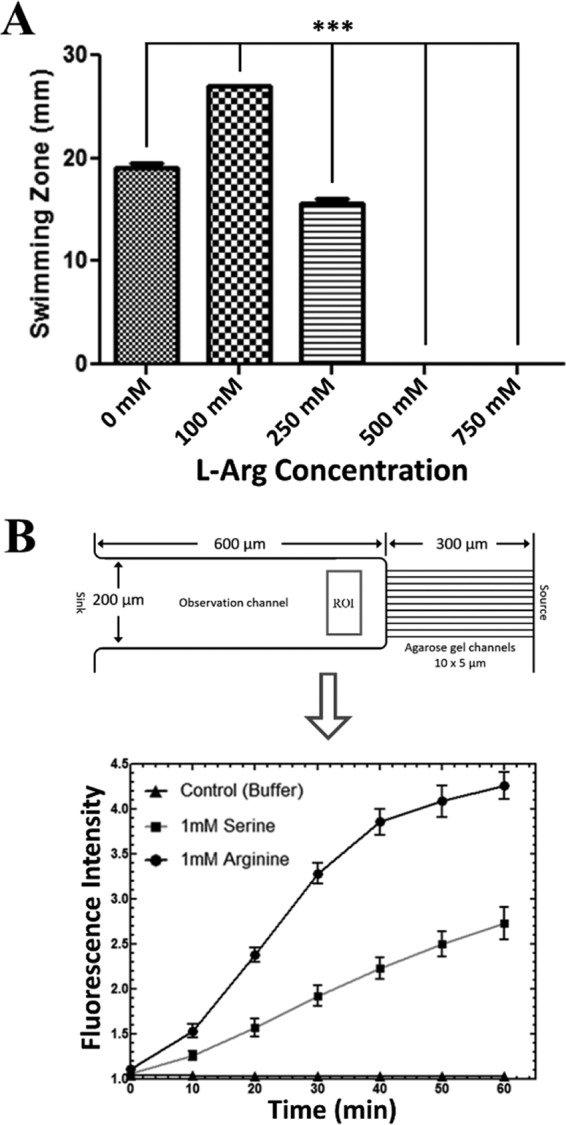
l-Arg is a potent chemoattractant for *P. aeruginosa* but reduces swimming motility at high concentrations. (A) Standard tryptone swim plates were supplemented with the indicated concentration of l-Arg. PAO1 swimming motility was significantly increased in the presence of 100 mM l-Arg by one-way ANOVA with Bonferroni’s multiple comparison posttest. ***, *P* < 0.001 (*n* = 6 to 9 replicates/group). PAO1 swimming motility was significantly reduced in the presence of 250, 500, and 750 mM l-Arg by one-way ANOVA with Bonferroni’s multiple comparison posttest. ***, *P* < 0.001 (*n* = 6 to 9 replicates/group). (B) Evaluation of PAO1-GFP swimming-mediated chemotaxis was carried out in an agarose gel-based two-layer microfluidic device. The cell density profile of PAO1-GFP was measured by the fluorescence intensity within the region of interest (ROI) in the observation well for the response toward the indicated concentration of the positive control, l-Ser, or l-Arg. The negative control consisted of buffer alone (1× PBS supplemented with 5 mM MgSO_4_ and 15 µM EDTA).

We next set out to determine the impact of reduced arginine levels on *P. aeruginosa* physiology during burn wound infection. Of course, arginine is critical for protein synthesis in all organisms; however, in *P. aeruginosa* amino acids often serve as cues that regulate numerous phenotypes, including motility (20). In addition, it is well established that flagella are crucial for *P. aeruginosa* to enter the bloodstream and cause sepsis in thermally injured mice (24, 25); the 50% lethal dose (LD_50_) of nonflagellated *P. aeruginosa* mutants can be as much as 10^5^ times greater than that of the wild-type strain in thermally injured mice. To test whether arginine levels impacted *P. aeruginosa* motility, we compared the swimming motilities of *P. aeruginosa* cells when grown on standard swimming plates supplemented with various concentrations of l-Arg (100, 250, 500, and 750 mM) (Fig. 2A). These concentrations fall within the range of l-Arg that have been administered topically to patients (26, 27). *P. aeruginosa* exhibited a small, but significant, increase in swimming motility when the medium was supplemented with 100 mM l-Arg; however, at higher concentrations, swimming motility was significantly reduced (Fig. 2A). To further test the impact of l-Arg on *P. aeruginosa* motility, we examined *P. aeruginosa* chemotaxis toward l-Arg using a diffusion-based microfluidic device (28). Analogous to previous reports evaluating *P. aeruginosa* chemotaxis, *P. aeruginosa* demonstrated a very robust chemotactic response toward l-Arg; however, the *P. aeruginosa* response toward l-Arg was nearly double in magnitude at every time point evaluated compared to that of l-Ser, a commonly used *P. aeruginosa* chemoattractant (29, 30) (Fig. 2B). Collectively, these data suggest that the reduced arginine levels in burns could trigger a response in swimming motility as a means for *P. aeruginosa* to seek out arginine via chemotaxis.

### l-Arg supplementation reduces *P. aeruginosa* spread and sepsis in thermally injured mice.

**FIG 3:**
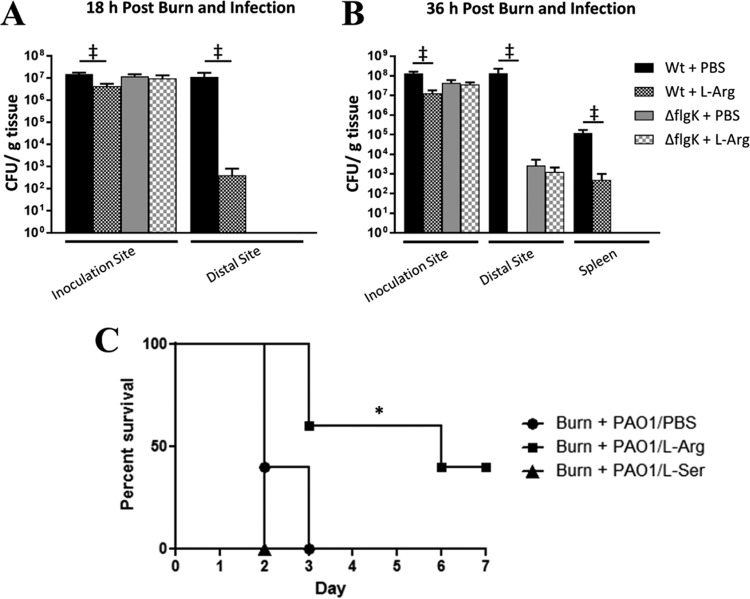
l-Arg supplementation reduced *P. aeruginosa* spread and mortality in thermally injured mice. Two groups of mice received third degree scald burns and were then inoculated with approximately 10^2^ CFU of the PAO1 (Wt) or *flgK* mutant combined with either 1× PBS or 0.125 g/ml l-Arg. Bacterial spread was determined in burned mice by quantifying the bacterial load (measured as CFU per gram of tissue) at defined locations. (A) Mean bacterial loads at 18 h at the inoculation and distal sites. ‡, *P* ≤ 0.05, one-tailed Mann-Whitney test (*n* = 3 to 5 mice/group). (B) Mean bacterial loads at 36 h at the inoculation and distal sites, as well as in the spleen. ‡, *P* ≤ 0.05, one-tailed Mann-Whitney test (*n* = 3 to 5 mice/group). It is worth noting that *P. aeruginosa* was cultured from the spleen of only one wild-type PAO1-infected l-Arg-treated mouse at 36 h; all other l-Arg-treated mice showed no signs of systemic infection. (C) Three groups of mice were subjected to a third degree scald burn and then immediately inoculated with approximately 10^2^ CFU of PAO1 combined with either 0.125 g/ml l-Arg (Burn + PAO1/l-Arg), 0.125 g/ml l-Ser (Burn + PAO1/l-Ser), or 1× PBS (Burn + PAO1/PBS). Supplementation of l-Arg in PAO1-infected burned mice significantly prolonged animal survival compared to control mice and mice that received l-Ser. *, *P* < 0.05, log-rank (Mantel-Cox) test (*n* = 5 mice/treatment group).

Based on the low levels of arginine present in burn wounds and our observation that low arginine levels trigger *P. aeruginosa* motility, we hypothesized that addition of l-Arg to the burn wound might attenuate the spread of *P. aeruginosa* in burned mice. To test this, we inoculated burned mice with *P. aeruginosa* cells mixed with either phosphate-buffered saline (PBS) or 0.125 g/ml l-Arg. This concentration of arginine equates to approximately 600 mM l-Arg HCl in each bolus injection or 1 g/kg of body weight per mouse and was previously shown to be safe for topical application in both rodents and humans (26, 31). In control mice (PBS treated), *P. aeruginosa* colonized and spread rapidly through the burn wound from the inoculation site to the perimeter of the wound by 18 h postburn and postinfection (Fig. 3A); however, addition of l-Arg resulted in slightly fewer *P. aeruginosa* cells at the inoculation site and over a 10,000-fold decrease in *P. aeruginosa* at the distal wound site. This response was magnified at later time points in infection when virtually no *P. aeruginosa* cells were found at the distal wound site in the l-Arg-treated group compared to 10^8^ cells found in PBS-treated mice (Fig. 3B). It is curious that bacteria in the wild-type group treated with l-Arg were able to spread to the distal site at 18 h but were not recoverable from the same site at 36 h; however, the two time points represent two different animal groups, and the inherent variability associated with *in vivo* models may partially account for this. Additionally, systemic spread of *P. aeruginosa* to the spleen was reduced over 100-fold by the addition of l-Arg (Fig. 3B).

For comparison, we also tested an avirulent *P. aeruginosa flgK* mutant that is defective in flagellum production and, subsequently, swimming motility. Because of this defect, the *flgK* mutant is unable to efficiently spread throughout the burn wound or cause sepsis, yet is still able to grow within the wound. We saw that l-Arg treatment does not significantly affect *P. aeruginosa* growth in the burn wound as there was no significant difference in bacterial loads at any sites between the PBS- or l-Arg-treated *flgK* mutant groups. Interestingly, the *flgK* mutant was able to reach the distal site at 36 h sans swimming motility. This is likely attributed to the fact that *P. aeruginosa* can facilitate other modes of motility beyond swimming (e.g., twitching). However, these other modes of motility are not as efficient as swimming, and it subsequently takes a much longer time for *P. aeruginosa* to disseminate when it lacks the ability to swim. The *flgK* mutant is also completely attenuated in burned mice, whereas a *pil* mutant, which is unable to twitch, remains fully virulent and causes a lethal infection in burned mice (unpublished results). Therefore, we concluded that the reduced spread of *P. aeruginosa* observed in the l-Arg-treated wild-type group is attributed to decreased swimming motility and not growth inhibition.

Based on these data, we hypothesized that l-Arg application to *P. aeruginosa* burn wound infections would increase animal survival. To test this hypothesis, we treated burned mice with l-Arg in the form of a bolus injection mixed with the bacterial inoculum followed by a second injection of either l-Arg or PBS directly into the burn wound at 24 h postinfection. Control mice (PBS treated) exhibited signs of sepsis by 48 h, and all became moribund by 72 h postburn and postinfection (Fig. 3C); however, a significant number of l-Arg-treated mice exhibited minimal signs of sickness and remained healthy up to the end of the study (Fig. 3C). It is worth noting that supplementation of l-Ser at the same concentration in burned and infected mice did not impart any advantage in terms of survival or animal health, suggesting that the effect of l-Arg has some degree of specificity (Fig. 3C). These results suggest that arginine may represent a useful tool to slow or prevent the dissemination of *P. aeruginosa* infection to the bloodstream in severely burned patients when applied locally into the burn wound.

## DISCUSSION

Here we report that administration of l-Arg hydrochloride significantly prolonged animal survival and reduced *P. aeruginosa* sepsis in burned mice. To our knowledge, this is the first demonstration that l-Arg can benefit animal survival by effectively reducing the systemic spread of this common nosocomial pathogen. The mechanism of this response is likely mediated by reversing the paucity of available arginine in the burn wound caused by exaggerated arginase production by MDSCs, which subsequently reduces *P. aeruginosa* motility. l-Arg is already frequently used as a dietary supplement to resuscitate burned individuals with cutaneous and lung injuries (32–36). Therefore, the development of an arginine-based topical treatment has the potential to both aid in patient recovery as well as reduce the spread of aggressive multidrug-resistant bacterial strains, like those of *P. aeruginosa*.

Previous reports have demonstrated that l-Arg reduced *P. aeruginosa* swarming motility and enhanced biofilm formation, suggesting that arginine represents an environmental cue that influences *P. aeruginosa* to favor a sessile, biofilm-forming lifestyle over a more active, motile one (20). However, up until now, this hypothesis had not previously been tested *in vivo*. Our data demonstrated that arginine potently inhibited *P. aeruginosa* swimming motility, and this likely resulted in the attenuation of sepsis *in vivo*. Although the concentrations of arginine evaluated here greatly exceed the basal levels of arginine in nonburned tissue, these concentrations are therapeutically administered in humans and have been proven to be safe. Furthermore, this is the first demonstration, to our knowledge, that these concentrations of arginine have a positive effect *in vivo* by negating the rapid dissemination of *P. aeruginosa* into the bloodstream via reduced swimming motility.

Given the strong chemotactic response *P. aeruginosa* displayed toward modest concentrations of l-Arg, it is possible that the aggressive dissemination of *P. aeruginosa* in burn wounds is driven by its need for this substrate when arginase production is elevated following MDSC recruitment. This finding would suggest a new role for MDSCs during infection: that the consequence of MDSC recruitment is not simply restricted to the classical obstruction of efficient activation of T cells and antimicrobial production, but that they also play a significant role in modulating bacterial behavior by limiting the availability of key nutrients, such as arginine, in the burn wound environment. Pharmacological options are available that can significantly decrease the number of MDSCs *in vivo* (37, 38); however, the negative physiological consequences associated with the use of chemotherapeutic drugs render them nonideal treatment options in burn patients. In contrast, the safety of concentrated doses and the potential wound-healing benefits of arginine make it an attractive alternative to counter the effects of exaggerated arginase production by MDSCs and subsequently deter *P. aeruginosa* motility into the surrounding tissue and bloodstream.

We are now in an era of very limited therapeutic options for infections caused by multidrug-resistant bacteria such as *P. aeruginosa*, and finding alternative treatments is imperative. Our data suggest that in addition to bactericidal agents, vaccines, and antivirulence approaches, modulating bacterial phenotypes by altering the infection microenvironment may be a promising method to halt sepsis. The effectiveness we observed here with therapeutic concentrations of arginine could potentially circumvent the complications associated with active and passive immunization strategies while still lowering the incidence of sepsis. Conventional antibiotics or other means of antimicrobial treatment will likely still be required to effectively clear the infection, but the use of arginine to modulate *P. aeruginosa* behavior and stymie systemic spread may prove to be a valuable component in future combinatorial therapies.

## MATERIALS AND METHODS

### Bacterial strains and growth conditions.

The *P. aeruginosa* strain PAO1 (39), PAO1 containing the green fluorescent protein (GFP)-expressing plasmid pMRP9-1 (40), and the PA14 *flgK* transposon mutant (41) have been described previously (40). For general growth of *P. aeruginosa*, strains were grown aerobically in Luria-Bertani (LB) broth at 37°C, with shaking at 200 rpm, unless otherwise noted. PAO1-GFP and *flgK* were grown aerobically at 37°C in LB broth supplemented with 300 μg/ml carbenicillin or 20 µg/ml tetracycline, respectively.

### Thermally injured mouse model of infection.

The thermally injured mouse model of infection was adapted from the burned-mouse model described by Stieritz and Holder (42–44). Burn experiments were conducted in adult female Swiss Webster mice (Charles River Laboratories, Inc.) weighing between 20 and 25 g. Mice were anesthetized by intraperitoneal injection of 0.4 ml of 5% sodium pentobarbital (Nembutal; Oak Pharmaceuticals, Inc.) at 5 mg/ml before their backs were shaved, and the hair was cleanly removed with a depilatory agent. Thermal injury was induced by placing an exposed area of the shaved skin (approximately 15% total body surface area) in a 90°C water bath for 10 s. This scald injury is nonlethal but induces a third-degree (full-thickness) burn. Where specified, nonburned mice served as controls, and their backs were shaved and treated with a depilatory agent in parallel with thermally injured mice before they were euthanized prior to collection of tissue. For survival experiments, mice were monitored for 7 days postburn and/or infection. At the specified end time point or in the event mice became moribund, mice were euthanized by intracardiac injection of 200 μl (390 mg/ml) Fatal-Plus (Vortech Pharmaceuticals, Ltd.). Animals were treated humanely and in accordance with protocol 96020 approved by the Institutional Animal Care and Use Committee at Texas Tech University Health Sciences Center in Lubbock, TX.

### Preparation of *P. aeruginosa* inoculum for challenge in thermally injured mice.

*P. aeruginosa* inocula for challenge in thermally injured mice were prepared as previously described (43, 44). For treatment experiments, approximately 10^2^ CFU PAO1 in 100 µl 1× PBS were combined with 200 µl of 0.125 g/ml of either l-Arg (l-Arg hydrochloride; Fisher BioReagents) or l-Ser (Fisher BioReagents) prepared in 1× PBS (pH 7.1 to 7.2) before inoculation into burned mice. Mice received the mixture of PAO1 cells plus l-Arg as a 300-µl bolus injection directly into the burn wound following thermal insult. Control mice received a 300-µl bolus injection of a mixture of PAO1 plus 1× PBS (approximately 10^2^ CFU of PAO1 combined with 200 µl 1× PBS) directly into the burn wound following thermal insult. Mixtures containing PAO1 cells plus l-Arg and PAO1 plus 1× PBS were prepared immediately prior to inoculation.

### Immunofluorescence imaging of MDSCs.

Immunofluorescence imaging of MDSC within burned and nonburned tissue was performed on frozen tissue sections. Briefly, tissue from thermally injured mice encompassing both the burn wound and the intact tissue bordering the burn was resized to approximately 1 cm by 0.5 cm and placed into a Tissue-Tek vinyl specimen Cryomold (Sakura Finetek) containing Cryomatrix optimum cutting temperature (OCT) compound (Thermo Fisher Scientific) and then placed into a freezer at −80°C to solidify before sectioning. Tissue from nonburned mice was resected from the center of the dorsum and prepared in parallel with burned tissue. Frozen OCT-embedded samples were sectioned using an OTF5000 cryostat (Bright Instrument Co., Ltd.) to a thickness of 4 to 6 µm and then directly transferred to Superfrost Plus microscope slides (Thermo Fisher Scientific) and stored at −80°C. Tissue samples were cut and oriented so that the burn wound-intact tissue interface could be best visualized. Tissue sections were incubated with fluorescein isothiocyanate (FITC)-labeled rat anti-mouse CD11b/Mac-1 antibody (Southern Biotech) (1:100 anti-CD11b, 1× PBS, 2% goat serum) and phycoerythrin (PE)-labeled rat anti-mouse Gr-1/Ly-6G antibody (Southern Biotech) (1:75 anti-Gr-1, 1× PBS, 2% goat serum). Each section was mounted with ProLong Gold antifade reagent (Molecular Probes) supplemented with DAPI (4′,6′-diamidino-2-phenylindole) to stain DNA. Mounted slides were visualized by epifluorescence microscopy with a Nikon Eclipse 80i microscope (Nikon), and images were captured with a Nikon DS-Fi1 camera (Nikon) and analyzed with the NIS Elements program (version 3.00 SP7; Nikon, Japan).

### ELISA for measurement of l-Arg and arginase.

Mouse l-Arg and arginase I concentrations were measured in tissue homogenates via commercially available enzyme-linked immunosorbent assay (ELISA) kits (MBS2600680 and MBS745422; MyBioSource) as per the manufacturer’s instructions. Briefly, approximately 100 mg of tissue was resected from the center of the wound in burned mice at 24 h postinsult (with or without infection) or from the center of the dorsum in noninjured mice. Tissue samples were placed into Precellys 2.8-mm steel bead kit (Peqlab) homogenizing tubes filled with 1 ml Pierce radioimmunoprecipitation assay (RIPA) buffer (Thermo Fisher Scientific) and then processed using a dedicated tissue homogenizer (Bertin Technologies). Homogenates were centrifuged, and the supernatants were collected from each sample. Aliquots of supernatants were then transferred to ELISA plates to measure l-Arg and arginase. Concentrations are normalized to the amount of tissue.

### Swimming motility assay and l-Arg-supplemented plates.

The swimming motility assay was adapted from Deziel et al. (45) and conducted on standard tryptone agar plates. For swim plates supplemented with l-Arg, l-Arg HCl (Fisher BioReagents) was added to standard tryptone swim medium to attain the specified concentration and adjusted to pH 7.1 to 7.2 before agar was added and the complete medium was autoclaved. l-Arg-supplemented swim plates were allowed to dry overnight at room temperature before inoculation.

### Chemotaxis in a microfluidic chip.

Evaluation of swimming-mediated chemotaxis was carried out in an agarose gel-based two-layer microfluidic device described previously (28) with the following modifications. PAO1-GFP cells were cultured in standard LB supplemented with carbenicillin to an optical density at 600 nm (OD_600_) of 1.0. Cells were harvested by centrifugation (300 × *g* for 4 min), washed in chemotaxis buffer (CB) (1× PBS supplemented with 5 mM MgSO_4_ and 15 µM EDTA), and then readjusted in CB to an OD_600_ of 0.2. CB was added to the source wells, and CB-washed PAO1-GFP cells were added to the sink wells. The cell density profile in the observation channel was incubated for 30 min, at which time CB in the source well was removed and replaced with 30 µl of the indicated chemoattractant (1 mM l-Arg in CB), positive control (1 mM l-Ser in CB), or CB alone as the negative control. Fluorescent images were acquired at 10-min intervals for 1 h. Cell density was measured by the fluorescence intensity within the region of interest (ROI) and was normalized to the background.

### Processing of tissue to determine local and systemic bacterial spread.

For experiments where bacterial spread was evaluated in burned mice, animals were euthanized at 18 or 36 h postinfection, and approximately 80 to 100 mg tissue was harvested from multiple sites, including tissue from the inoculation (center of wound) and distal/periphery (~3 cm from inoculation site) sites, as well as spleens where noted. Harvested tissue was then placed in Precellys 2.8-mm steel bead kit homogenizing tubes filled with 1× PBS and homogenized using a dedicated tissue homogenizer. Burn wound and spleen homogenates were then serially diluted (10-fold), plated on *Pseudomonas* isolation agar, and incubated overnight at 37°C before CFU were counted and the bacterial load (CFU per gram of tissue) was calculated for each sample.
