# Supplementary material for: Arginine Is a Critical Substrate for the Pathogenesis of Pseudomonas aeruginosa in Burn Wound Infections
Source: mBio. 2017 Mar 14;8(2):e02160-16. doi: 10.1128/mBio.02160-16 (PMC5350470; doi:10.1128/mBio.02160-16)
Supplement: FIG S1 [file mbo001173211sf1.pdf]

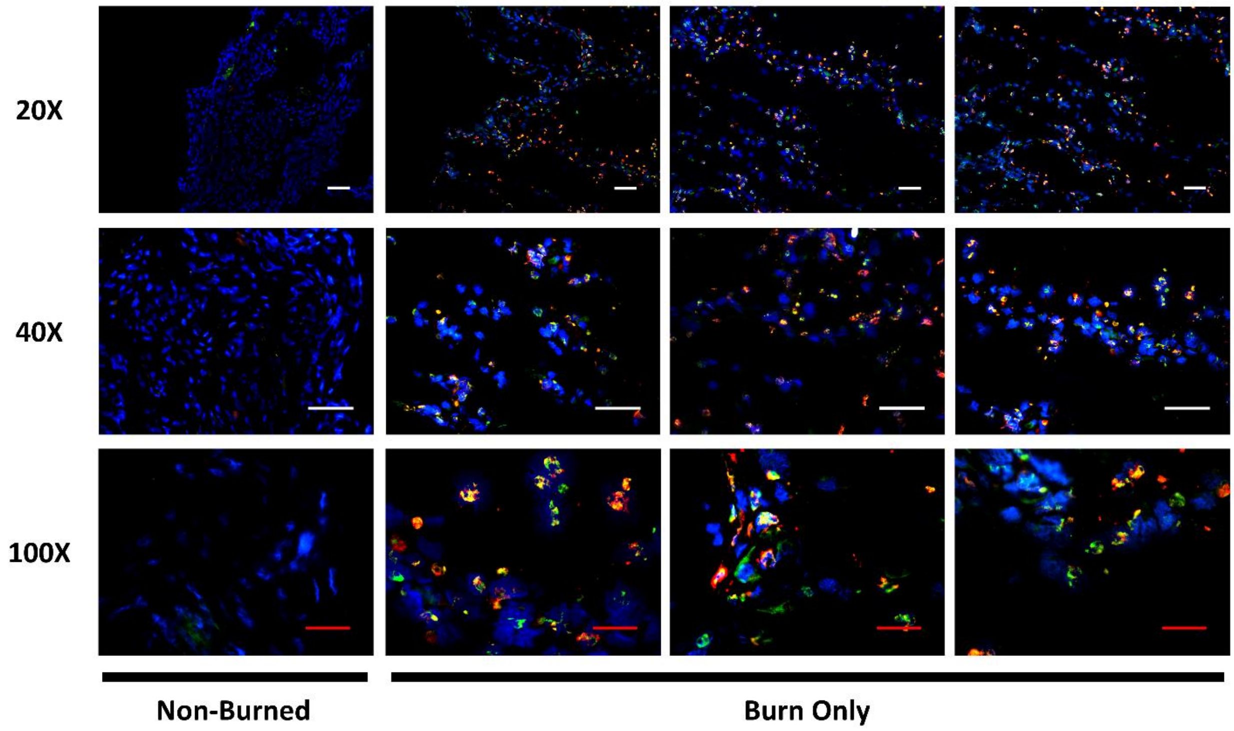

<u>Figure S1</u>. MDSC recruitment in thermally injured mice. Tissue was harvested from burn only mice 24h post-thermal insult or non-burned mice. Tissue samples were prepared for direct immunofluorescence microscopy with FITC-labeled anti-CD11b (green) and PE-labeled anti-Gr-1 antibodies (red). Host cell nuclei were counterstained with DAPI (blue). A large number of MDSCs co-expressing both CD11b and Gr-1 are recruited to the burn-intact tissue interface. No MDSCs were observed in the tissue from the dorsum of non-burned mice. Sections were visualized via a Nikon Plan Fluor 20X/0.75, 40X/1.30 Oil, and 100X/1.30 Oil objectives. White bars represent 50µm. Red bars represent 20 µm.
